# Cellular Redox Status Regulates Emodin-Induced Radiosensitization of Nasopharyngeal Carcinoma Cells In Vitro and In Vivo

**DOI:** 10.1155/2013/218297

**Published:** 2013-04-16

**Authors:** Huaxin Hou, Danrong Li, Daohai Cheng, Li Li, Ying Liu, Yi Zhou

**Affiliations:** ^1^College of Pharmacy, Guangxi Medical University, Nanning 530021, China; ^2^Department of Basic Research, Guangxi Institute for Cancer Research, 71 Hedi Road, Nanning 530021, China

## Abstract

Here, we report that regulation of cellular redox status is required for radiosensitization of nasopharyngeal carcinoma (NPC) cells by emodin. We evaluated emodin's radiosensitivity-enhancing ability by using NPC cells in vitro and xenografts in vivo. A clonogenic assay was performed to evaluate NPC cell survival and to determine dose modification factors. Flow cytometry, western blot analysis, and in vivo radiation-induced tumor regrowth delay assays were performed to characterize emodin's effects. Exposure of CNE-1 NPC cells to emodin enhanced their radiosensitivity. HIF-1*α* expression significantly increased under hypoxic conditions but did not change after treatment with emodin alone. Emodin downregulated mRNA and protein expression of HIF-1*α*. Cells exposed to radiation and emodin underwent significant cell cycle arrest at the G_2_/M phase. The percentage of apoptotic cells and reactive oxygen species (ROS) levels were significantly higher in the group exposed to emodin and radiation hypoxic group than in the other groups. Compared to the CNE-1 xenografts exposed to radiation alone, CNE-1 xenografts exposed to radiation with emodin showed significantly enhanced radiation effects. Our data suggest that emodin effectively enhanced the radiosensitivity of CNE-1 cells in vitro and in vivo. The mechanism appears to involve ROS generation and ROS-mediated inhibition of HIF-1*α* expression.

## 1. Introduction

One of the most common types of head and neck cancer is nasopharyngeal carcinoma (NPC), a highly metastatic, invasive, and malignant cancer that shows a marked geographic and racial distribution. Statistics indicate that approximately 80 percent of NPC patients worldwide are Chinese, and there is a marked prevalence of NPC in southern China. The incidence rate is typically 2 to 3 times higher in men than in women, both in developing and developed countries [1].

The majority of patients diagnosed with NPC undergo radiation therapy, but some patients receiving radiation present with local tumor recurrence and distant metastases after radiotherapy. This is believed to be caused by radioresistance, and the majority of these patients exhibit tumor recurrence and metastasis within 1.5 years after completion of the treatment regimen. Many evidence supports the idea that hypoxia is an important factor in the growth of solid tumors in humans. Hypoxic cancer cells may undergo a series of genetic and metabolic changes that enable them to survive and proliferate and to become more resistant to conventional therapies, including ionizing radiation and chemotherapy [2]. Tumor hypoxia has become an obstacle for both chemotherapy and radiotherapy. An increasing number of studies show that the bioreductive agent emodin (1,3,8-trihydroxy-6-methylanthraquinone) can reverse multidrug resistance or enhance the cytotoxicity of chemotherapeutic drugs [3–6]. However, few studies have investigated whether emodin alters nasopharyngeal carcinoma cell proliferation in vitro. Here, we examined the radiosensitization effects of emodin on CNE-1 cells. We also investigated possible signaling mechanisms underlying radiosensitization effects.

## 2. Materials and Methods

### 2.1. Reagents

Emodin (98% purity) was extracted from the roots of *Polygonum multiflorum* (PM) Thunb, and its physical and chemical properties were consistent with previously published reports [7]. Stock solutions of emodin (500 *μ*M) in DMSO were diluted with cell culture medium such that the final concentration of DMSO was less than 0.1%. For in vivo studies, emodin was dissolved in 0.9% sterile saline to a final concentration of 2.75 mg/mL.

### 2.2. Cell Culture

The human nasopharyngeal carcinoma cell line CNE-1 was obtained from the Guangxi Institute for Cancer Research. Cells grown under normoxic conditions were cultured in RPMI 1640 medium (Gibco, Grand Island, NY, USA) containing 10% (v/v) heat-inactivated newborn bovine serum (NBS) (Gibco, Grand Island, NY, USA), 100 *μ*g/mL streptomycin (Lukang Pharmaceutical, Shandong, China), and 100 IU/mL penicillin (Lukang Pharmaceutical, Shandong, China) at 37°C in a humidified atmosphere with 5% CO_2_. All cultures routinely tested negative for contamination by mycoplasma or fungi. All cell lines were discarded after 3 months, and new lines were obtained from frozen stocks. In vitro hypoxic experiments were performed in a temperature and humidity-controlled hypoxic chamber set at 95% N_2_/5% CO_2_ or 95% air/5% CO_2_ (COY laboratory equipment, Grass Lake, MI, USA). The apparatus contained a separate access chamber, as well as two pairs of work gloves, enabling manipulation of cultures in a hypoxic environment.

### 2.3. MTT Assay

The inhibitory rate of CNE-1 cells was lower than 10% at the concentration of emodin that was selected as a noncytotoxic dose. The MTT (St Louis, MO, USA) assay was performed as described previously, with minor modifications [8]. Briefly, CNE-1 cells were harvested with trypsin and resuspended to a final density of 1 × 10^5^ cells/mL. Aliquots of 100 *μ*L from each cell suspension were distributed evenly into Costar 96-well cell culture plates. After the cells had been incubated for 24 h, designated wells were treated with different concentrations of emodin. After incubation for 48 h, 20 *μ*L MTT solution (5 mg/mL) was added into each well and incubated at 37°C in a 5% CO_2_ atmosphere for 4 h. Then, the solution was removed from the wells and formazan crystals in each well were solubilized in 200 *μ*L dimethyl sulfoxide (DMSO). The reduction of MTT was quantified by absorbance at a wavelength of 490 nm using a Multiskan MK3 (Thermo Fisher Scientific, Waltham, MA, USA). Three wells were quantified for each condition. The percent inhibition was calculated as follows: % inhibition = [1 − (mean *A* of sample/mean *A* of control)] × 100%. 

### 2.4. Clonogenic Cell Survival Assay

The CNE-1 cells were trypsinised and counted, and the appropriate number of cells was plated in 60 mm dishes and allowed to attach for 24 h. After treating the cells with either 3.9 mg/L emodin or 7.8 mg/L emodin for 24 h, the cells were irradiated (2, 4, 6, 8 Gy) under hypoxic or normoxic conditions and incubated for additional 10–14 days. Colonies were fixed with methanol/acetic acid (3 : 1) and stained with crystal violet. Colonies with >50 cells were scored and cell survival determined after correcting for the plating efficiency and for cytotoxicity caused by emodin alone. Survival curve data were fit using a linear-quadratic model according to Albright [9, 10]. Survival curves for each colony were repeated 2–3 times. The dose modification factor (DMF) was calculated as the ratio of radiation doses at the 10% survival rate (control radiation dose divided by the emodin-treated radiation dose). DMF values >1 indicated enhancement of radiosensitivity.

### 2.5. Annexin V-Propidium Iodide Assays for Apoptosis

The CNE-1 cells were divided into eight groups: group A (control cells), group B (cells cultured under hypoxic conditions), group C (cells treated with 3.9 mg/L emodin only), group D (cells treated with 2 Gy only), group E (cells treated with 3.9 mg/L emodin under hypoxic conditions), group F (cells treated with 2 Gy under hypoxic conditions), group G (cells treated with 3.9 mg/L emodin and 2 Gy), and group H (cells treated with 3.9 mg/L emodin and 2 Gy under hypoxic conditions). All cells were incubated at 37°C for 24 h. For annexin V-propidium iodide (PI) assays, cells were stained and evaluated for apoptosis using flow cytometry according to the manufacturer's protocol. Briefly, 1 × 10^6^ cells were collected and stained with 5 *μ*L annexin V-fluorescein isothiocyanate (FITC, BD Biosciences Pharmingen, San Jose, CA, USA) and 5 *μ*L PI in 1x binding buffer for 15 min at room temperature in the dark. The apoptotic cells were quantified using a FACScan Cytometer (BD Biosciences).

### 2.6. ROS Detection

Cells were divided into the same groups described above. The levels of ROS in cells were measured using 2′,7′-dichlorofluorescin diacetate (DCFH-DA), an oxygen-sensitive fluorescent probe (Beyotime Company, Jiangsu, China). Cells were collected according to the manufacturer's instructions. DCFH-DA was added at a final concentration of 10 *μ*M to each sample and incubated at room temperature for 15 minutes. Cells were then collected and washed with serum-free medium. Samples were analyzed by flow cytometry on a FACSCalibur. The average intensity of DCF fluorescence corresponded to intracellular ROS levels. The relative fluorescence intensity was averaged across all experiments.

### 2.7. Real-Time Quantitative PCR

Total RNA from cells was extracted using Trizol reagent (Invitrogen, Carlsbad, CA, USA) according to the manufacturer's recommendations. First strand cDNA synthesis (MBI Fermentas, Hanover, MD, USA) was carried out using 3 *μ*g of total RNA with M-MuLV reverse transcriptase and oligo-(dT)_18_ as a primer. Real-time quantitative PCR primers (TaKaRa Biotech, Dalian, China) for HIF-1*α* (302 bp) and for the housekeeping gene *β*-actin (247 bp) were selected based on published sequences [11]. The primers for genes were as follows: HIF-1*α*: sense: 5′-AGC CGC TGG AGA CAC AAT-3′ and antisense: 5′-TCG GAA GGA CTA GGT GTC TGA-3′; *β*-actin: sense: 5′-AAC TCC ATC ATG AAG TGT GA-3′ and antisense: 5′-ACT CCT GCT TGC TGA TCC AC-3′. The optimal reaction mixture (25 *μ*L total volume) contained 2.5 *μ*L buffer (10x Mg^2+^-free), 2.5 *μ*L Mg^2+^ (25 mM), 2.0 *μ*L dNTP (2.5 mM), 0.5 *μ*L sense primer (10 *μ*M), 0.5 *μ*L antisense primer (10 *μ*M), 0.2 *μ*L rTaq DNA polymerase (5 U/*μ*L), 1.0 *μ*L SYBR Green (10x), 2.0 *μ*L template, and 13.8 *μ*L ddH_2_O. Samples were first denatured at 94°C for 5 min followed by a maximum of 45 PCR cycles. Each PCR cycle consisted of denaturation at 94°C for 30 s, annealing at 60°C for 30 s, and extension at 72°C for 30 s. This was followed by a final extension at 72°C for 10 min. All reactions were carried out in the iCycler Thermal Cycler (Bio-RAD). Each PCR amplification reaction was performed in triplicate wells. The purity of the amplified PCR products was verified by melt curve analysis. Relative quantification was accomplished by quantification of the threshold cycle (*C*_*t*_) values and use of the standard curve. The standard curves were established as described previously [12]. Relative expression was then calculated as follows:

(1) F=10ΔCT,T/AT−ΔCT,R/AR,

where *F* refers to relative expression index; Δ*C*_*T*,*T*_ = *C*_*t*_ (sample, HIF-1*α*) −  *C*_*t*_ (control, HIF-1*α*); Δ*C*_*T*,*R*_ = *C*_*t*_ (sample, *β*-actin) −  *C*_*t*_ (control, *β*-actin); *A*_*T*_ = the slope of the standard curves for HIF-1*α*; *A*_*R*_ = the slope of the standard curves for *β*-actin. 

### 2.8. Western Blot Analysis

Protein levels of HIF-1*α* in the cells were estimated by western blotting. After 48 h of stimulation, whole cell extracts were prepared with cell lysis buffer. Protein concentrations were quantified with the enhanced BCA protein assay (Pierce Biotechnology, Rockford, IL, USA). The housekeeping protein GAPDH was used as an internal control. Proteins were loaded (20 *μ*g) and separated by 7.5% (HIF-1*α*) and 12.0% (GAPDH) SDS-polyacrylamide gel electrophoresis. Gels were subsequently transferred to PVDF membranes (Millipore, Billerica, MA, USA). HRP-conjugated monoclonal mouse anti-GAPDH and rabbit polyclonal antibody HIF-1*α* (Santa Cruz, CA, USA) diluted to 1/5,000 and 1/200, respectively, were incubated at 4°C overnight after blocking with 5% (w/v) nonfat dry milk powder in PBS for 2 h at room temperature. After washing with PBST buffer (PBS containing 0.05% Tween 20), the membrane for HIF-1*α* was incubated with an HRP-labeled goat anti-rabbit IgG diluted to 1/3,000 for 4 h at 4°C. The membranes were washed again, and the blots were visualized with SuperSignal West Pico Chemiluminescent Substrate (Pierce Biotechnology, Rockford, IL, USA). The luminescence densities of each band were calculated using the Bio-Rad Quantity One software. Optical density values were normalized to the values of GAPDH of each sample.

### 2.9. Xenograft Studies

Nude mice were inoculated (s.c) in both flanks with 5 × 10^6^ CNE-1 cells. The mice were randomly distributed into treatment groups, and treatment was started when the tumor size reached 80 mm^3^ at 10 days after inoculation. Mice bearing nasopharyngeal carcinoma were randomly divided into seven groups, with each group containing 6 animals: control, solvent only, emodin only, radiotherapy only, and combination of emodin and radiotherapy. The emodin group and combination group were divided into 2 subgroups according to the high dose (12 mg/kg) and low dose (4 mg/kg) of emodin administered. Emodin and radiation (2 Gy) were given once a day, 5 days a week; the total radiation dose was 10 Gy. Radiation was delivered directly to the tumor while shielding the rest of the animal. The tumor diameters of every group were measured on alternating days. Documenting the tumor volume of each individual mouse enabled the determination of the time required (in days) for a tumor to reach 2 times the starting tumor volume. The tumor growth delay was calculated. The enhancement factor (EF) was used to evaluate radiosensitive enhancement. All animal studies were conducted in accordance with the guidelines established by our University Committee on Use and Care of Animals.

### 2.10. Statistical Analysis

Results are presented as the mean ± SD $\left(\bar{x}\pm s\right)$. One-way analysis of variance (ANOVA) was used for statistical analysis. A *P* value <0.05 was considered to be statistically significant. All experiments were repeated three times.

## 3. Results

### 3.1. Cytotoxic Effects of Emodin on CNE-1 Cells

Emodin had a minor inhibitory influence on proliferation of CNE-1 cells. The IC_50_ for emodin was 465 *μ*g/mL. No cytotoxicity was observed in CNE-1 cells provided that the concentration of emodin was kept lower than 62.5 *μ*g/mL, where the inhibitory ratio was less than 10%. As shown in Table 1, neither 3.9 nor 7.8 *μ*g/mL emodin had significant cytotoxic effects on CNE-1 cells. On the basis of these data, we adopted a paradigm where treatment with 3.9 *μ*g/mL emodin was started 24 h before irradiation, as the standard protocol for radiation experiments.

### 3.2. Modulation of Radiation Resistance by Emodin In Vitro

**Figure 1:**
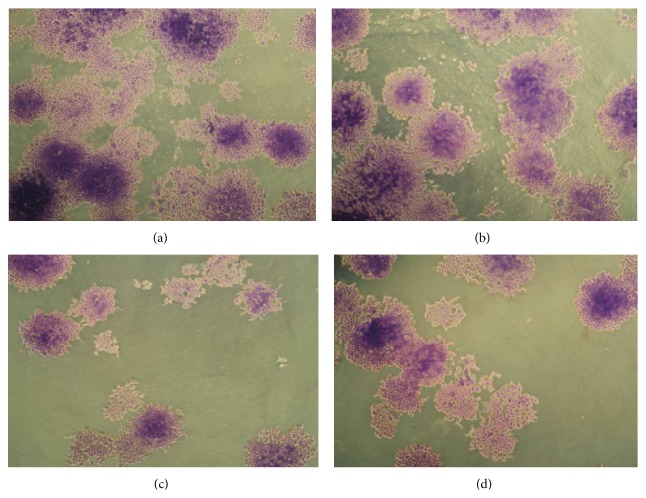
Effect of colonies formation on emodin in CNE-1 cells. Cell colonies morphological changes under inverted microscope (×50), fixed with paraformaldehyde 4% and stained with Giemsa. (a) Hypoxia group, (b) hypoxia + 2 Gy, (c) emodin (7.8 *μ*g/mL) + hypoxia + 2 Gy, and (d) emodin (3.9 *μ*g/mL) + hypoxia + 2 Gy.

**Figure 2:**
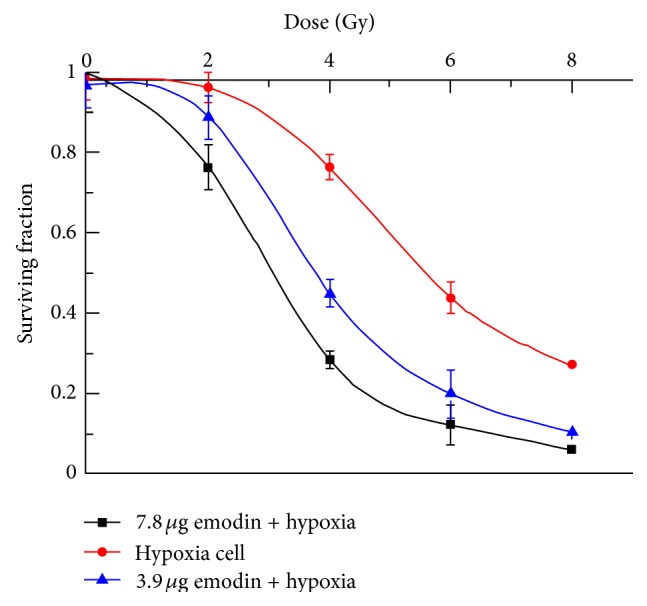
The survival curve of CNE-1 cells.

The colony numbers and fraction of CNE-1 cells surviving treatment with 3.9 *μ*g/mL or 7.8 *μ*g/mL emodin combined with radiation under hypoxic conditions are shown in Table 2 and Figure 1, respectively. The survival curve and the sensitization enhancement ratio for each group are shown in Table 3 and Figure 2. CNE-1 cells exposed to emodin at noncytotoxic concentrations (3.9 *μ*g/mL and 7.8 *μ*g/mL) exhibited radiosensitive effects. Dose modification factors (DMF) were 1.436 and 1.832, respectively. Radiosensitivity of cells treated with 7.8 *μ*g/mL emodin was greater than that observed in the 3.9 *μ*g/mL emodin treatment group, demonstrating a dose-dependent effect of emodin.

A clonogenic assay was used to determine the effects of emodin on radiosensitivity. Cells were seeded as a single-cell suspension with a specified number of cells. After allowing cells time to attach, emodin or the control solution was added at specified concentrations and the plates were later irradiated. Ten to fourteen days after seeding, survival curves were generated after normalizing for the cytotoxicity contributed by treatment with emodin alone. Figure 2 shows cytotoxicity of emodin alone and the radiation dose enhancement factor (DEF) for CNE-1 cells with combined drug treatment and radioation. Data presented are the mean ± SE from at least three independent experiments.

### 3.3. Apoptosis Induction

**Figure 3:**
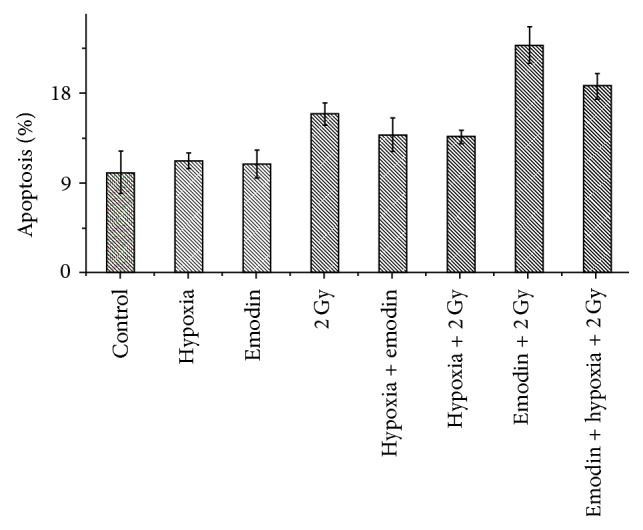
Apoptosis rate of CNE-1 cells treated with radiation or combined with emodin.

We examined the influence of emodin on radiation-induced apoptotic cell death under hypoxic conditions. As shown in Table 4 and Figure 3, the apoptosis ratio (%) of CNE-1 cells was 22.8 ± 1.8 in the group treated with emodin combined with radiation, 15.9 ± 1.1 in the group receiving radiation alone, 10.9 ± 1.4 in the emodin group, and 10.1 ± 2.1 in the control group. The apoptosis ratio in the group receiving emodin combined with radiation was significantly higher than that of all other groups (*P* < 0.05). In groups receiving emodin combined with radiation, a significant number of cells arrested in G_2_/M phase. 

### 3.4. Emodin-Induced Production of Reactive Oxygen Species (ROS)

**Figure 4:**
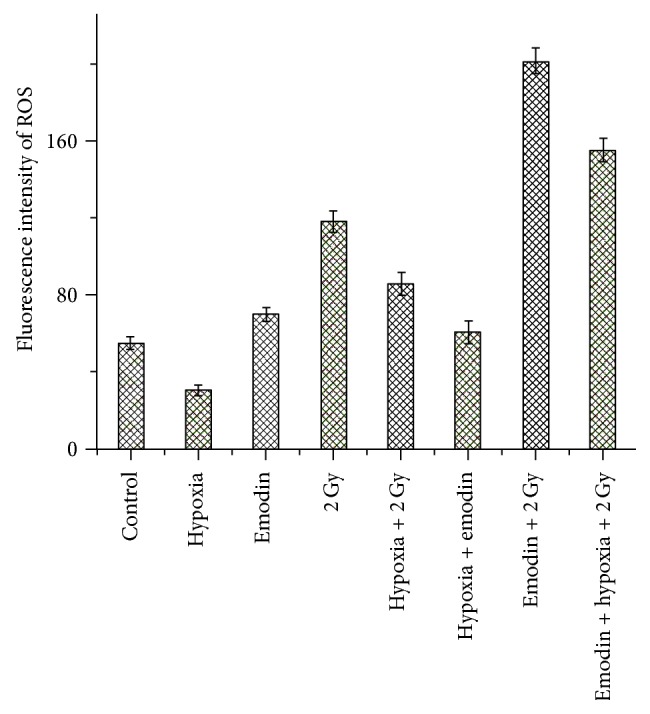
Fluorescence intensity of ROS in CNE-1 cell treated with radiation or combined with emodin.

Following exposure of CNE-1 cells to noncytotoxic concentrations of emodin (3.9 *μ*g/mL) or coadministration of emodin and radiation under hypoxic conditions for 24 h, the levels of ROS production were quantified using flow cytometry, monitoring fluorescence intensity. Compared to the control group, the relative content of ROS in CNE-1 cells determined at 24 h was 30.19 ± 2.69 in the hypoxic group, 117.71 ± 5.47 in the group exposed only to radiation, and 154.91 ± 6.25 in the group exposed to emodin combined with radiation (Table 5 and Figure 4). At 24 h, the relative amount of ROS in CNE-1 cells receiving emodin combined with radiation was significantly higher than those exposed to radiation alone, under hypoxic conditions (*P* < 0.05).

### 3.5. Effect of Emodin Treatment on HIF-1*α* mRNA Levels

**Figure 5:**
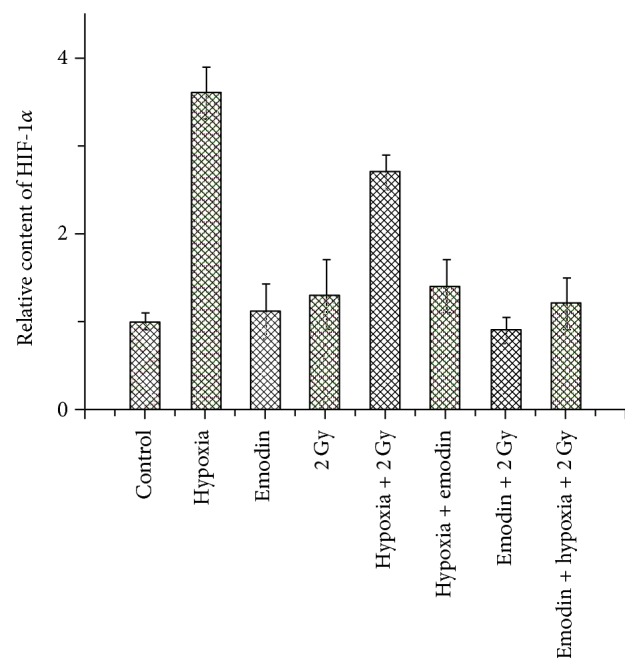
Changes of HIF-1*α* mRNA expression in CNE-1 treated with 2 Gy or combined with emodin.

It was previously reported that regulation of HIF-1 activity is primarily determined by the stability of the HIF-1*α* protein, which is sensitive to intracellular ROS. The results of mRNA quantification are depicted in Figure 5. Real-time quantitative PCR showed that HIF-1*α* mRNA levels detected in RNA extracts from the CNE-1 cells under hypoxic conditions were much greater (*P* < 0.01) than the levels in CNE-1 cells treated with 2 Gy or 2 Gy + emodin. The levels of HIF-1*α* mRNA in CNE-1 cells exposed to a combination of 2 Gy and emodin were markedly decreased (*P* < 0.01), when compared with CNE-1 cells exposed to hypoxia and radiation.

### 3.6. Effect of Emodin Treatment on the Levels of HIF-1*α* Protein

**Figure 6:**
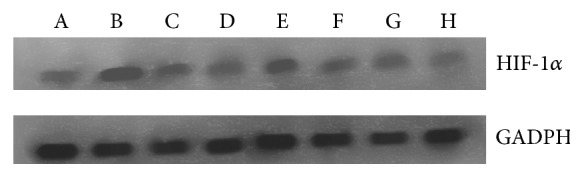
Changes in HIF-1*α* protein expression in CNE-1 cells treated with radiation alone or radiation combined with emodin. Group A (control cells), group B (cells cultured under hypoxic conditions), group C (cells treated with 3.9 mg/L emodin only), group D (cells treated with 2 Gy only), group E (cells treated with 3.9 mg/L emodin under hypoxic conditions), group F (cells treated with 2 Gy under hypoxic conditions), group G (cells treated with 3.9 mg/L emodin and 2 Gy), and group H (cells treated with 3.9 mg/L emodin and 2 Gy under hypoxic conditions).

Western blot assay showed that the expression of HIF-1*α* protein in CNE-1 cells was markedly (*P* < 0.01) higher than the level of the cells treated with radioation or emodin under hypoxia condition (Figure 6). The levels of HIF-1*α* protein in the CNE-1 cells with combination of radiation and emodin (3.9 mg/L) were significantly (*P* < 0.01) decreased compared with CNE-1 cells exposed to radiation alone. The result is similar as the data on effects of emodin on HIF-1*α* mRNA levels [11].

### 3.7. Emodin Enhances Radiation-Induced Tumor Growth Delay in CNE-1 Xenografts

For the CNE-1 xenograft model, the time required for tumors to double in volume from the initiation of treatment increased from 17.02 ± 1.12 days for control mice to 17.14 ± 0.63 days for solvent mice. Treatment with 2 Gy increased the time required to double tumor volume to 21.30 ± 1.14 days. However, in mice that received a combination of 2 Gy + emodin, the time for tumors to double increased to 24.10 ± 0.72 days for low dose emodin and 26.17 ± 0.64 days for high dose emodin. The absolute growth delays (the time in days for tumors in treated mice to double in volume minus the time in days for tumors to reach the same size in control mice) were 0.2 days for low-dose emodin alone, 0.40 days for high-dose emodin alone, 4.28 days for 2 Gy, 6.90 days for 2 Gy + emodin (4 mg/kg), and 9.15 days for 2 Gy + emodin (12 mg/kg). Thus, the combined treatment was more than the sum of the growth delays caused by individual treatments [10] (Table 6). 

## 4. Discussion

Selectively killing cancer cells without harming normal tissue is the primary goal in cancer therapy. Elevated oxidative stress and aberrant redox homeostasis are frequently observed in cancer cells compared to their normal cell counterparts. Hypoxia in tumors is generally associated with radioresistance, mainly because such therapies require adequate intratumoral oxygen to be maximally cytotoxic. Hypoxia may also reduce tumor sensitivity to treatment through one or more indirect mechanisms that include proteomic and genomic changes. These effects, in turn, can lead to increased invasiveness and metastatic potential, loss of apoptosis, and chaotic angiogenesis, thereby further increasing treatment resistance. HIF-1*α* is a heterodimer composed of a 120-kDa HIF-1*α* subunit and a 91–94-kDa HIF-1*β*/ARNT subunit. The HIF-1*α* subunit possesses a unique oxygen-dependent degradation domain (ODD) that controls protein stability and plays a crucial role in regulating the state of oxygen in tumor microenvironment [13]. HIF-1*α* expression is also associated with poor prognosis and resistance to radiation therapy in lung cancer, colon cancer, and cervical cancer [14, 15]. Xueguan et al. [16] reported that elevated expression of the HIF-1*α* gene in NPC cells correlated significantly with resistance to radiation therapy. This supported the hypothesis that inhibiting HIF-1*α* gene expression may overcome radiation resistance in NPC cells. These results confirm that HIF-1*α* plays a key role in the adaptive response to hypoxia, and it is not unreasonable to assume that HIF-1*α* may provide a target for improving the efficacy of radiation therapy as well. 

Under physiological conditions, small amounts of reactive oxygen species (ROS) are constantly produced in aerobic metabolism and have important roles in normal cell physiology, for example, in signal transduction pathways. However, excessive production of ROS in mitochondria has been recognized as a mediator of the apoptotic signaling pathway. Emodin is a type of natural anthraquinone with a molecular structure similar to that of (2,3-dimethoxy-1,4-naphthoquinone)DMNQ and mitochondrial ubiquinone, which are considered as endogenous ROS generators because of their properties of transferring electrons. Since the oxidation-reduction potential (ORP) is lower than that of the oxygen, these bioreductive drugs can be reduced to cytotoxic agents under hypoxic conditions, sensitizing the cells to x-irradiation [8, 15, 17, 18]. Here, we show that 24 h after treatment with emodin, ROS levels are significantly elevated compared to that which was observed in hypoxic conditions. In the group that received treatment combining emodin with radiation, ROS levels were also significantly higher than those in the group treated with radioation alone, indicating that emodin can induce ROS generation in CNE-1 cells, with the excessive amounts of ROS causing oxidative damage and apoptotic cell death. The results also show that emodin downregulates HIF-1*α* expression of mRNA and protein levels in CNE-1 cells. Overexpression and excessive activation of HIF-1*α* in radiation-resistant cells are associated with promotion of survival and prevention of apoptosis. Therefore, the mechanism underlying emodin-elevated radiosensitivity of NPC cells may likely involve ROS-induced apoptosis and inhibition of protein expression of either HIF-1*α* or a related gene.

In conclusion, modulation of the redox status of cancer cells to enhance cytotoxicity of radiation represents a viable therapeutic strategy, with HIF-1 considered as an important therapeutic target. Because emodin is a novel small molecule inhibitor of HIF-1, it may serve as an effective radiosensor to improve efficacy of radiation therapy in radiation-resistant cancer cells, particularly cells with upregulated HIF-1. Since emodin can effectively enhance the radiosensitivity of CNE-1 cells in vivo, emodin holds promise for future development as a novel class of radiosensitizing drugs for patients with nasopharyngeal carcinoma.

## Figures and Tables

**Table 1:** Inhibition rate of CNE-1 cell proliferation as a function of emodin concentration.

Emodin (*μ*g/mL)	OD value ($\overset{-}{x}\pm s$)	Inhibition rate (%)
0	0.843 ± 0.117	0
3.9	0.818 ± 0.073	—
7.8	0.788 ± 0.164	—
15.6	0.766 ± 0.060	0.91
31.25	0.743 ± 0.090	3.88
62.5	0.699 ± 0.049	9.57
125.0	0.558 ± 0.041	27.81
250.0	0.424 ± 0.023	45.14

**Table 2:** Cell survival rates, expressed as a fraction of control (*n* = 3, $\overset{-}{x}\pm s$).

Doses (Gy)	Fraction surviving			
	radiation	hypoxia + radiation	3.9 *μ*g/mL emodin + hypoxia + radiation	7.8 *μ*g/mL emodin + hypoxia + radiation
0	1.000 ± 0.000	0.983 ± 0.010	0.972 ± 0.022	0.941 ± 0.041
2	0.763 ± 0.034	0.962 ± 0.021	0.890 ± 0.025	0.843 ± 0.036
4	0.284 ± 0.020	0.764 ± 0.014	0.423 ± 0.019	0.370 ± 0.027
6	0.122 ± 0.019	0.438 ± 0.023	0.179 ± 0.030	0.162 ± 0.018
8	0.060 ± 0.004	0.272 ± 0.016	0.103 ± 0.040	0.080 ± 0.004

**Table 3:** Parameter of cell survival curve.

Emodin (*μ*g/mL)	*D*_ 0_		DMF
	Radiation alone	Hypoxia + radiation
0	1.672	2.762	—
3.9	1.347	1.923	1.436
7.8	1.021	1.508	1.832

**Table 4:** Effects of emodin and radiation on apoptosis and cell cycle progression of CNE-1 cells ($\overset{-}{x}\pm s$) %.

Groups	G_0_/G_1_	G_2_/M	*S*	SI	Apoptosis
Control	70.53 ± 1.34	10.21 ± 0.66	19.26 ± 0.86	—	10.1 ± 2.1
Hypoxia	72.16 ± 3.73	5.81 ± 0.15	22.03 ± 1.12	1.79	11.2 ± 0.8
Emodin	68.73 ± 0.67	7.41 ± 0.14	23.86 ± 1.16	1.34	10.9 ± 1.4
2 Gy	68.27 ± 3.34	14.50 ± 0.25	17.14 ± 0.64	0.68	15.9 ±1.1^⋆^
Hypoxia + emodin	68.91 ± 1.32	8.01 ± 0.21	23.12 ± 1.35	1.24	13.8 ± 1.7
Hypoxia + 2 Gy	69.27 ± 2.62	13.91 ± 0.17	16.81 ± 0.82	0.72	13.6 ± 0.7
Emodin + 2 Gy	67.24 ± 1.64	20.51 ± 1.02	12.19 ± 0.14	0.47∗	22.8 ± 1.8^*⋆*^
Emodin + hypoxia + 2 Gy	63.33 ± 1.79	21.14 ± 1.21	15.53 ± 0.32	0.43∗	18.7 ± 1.3^*⋆*^

SI (sensitivity index) = (experimental group G_0_G_1_/G_2_M)/(control group G_0_G_1_/G_2_M); ∗SI ≤ 0.5, indicated by the duration of G_2_M phase.

^⋆^ *P* < 0.05 compared with hypoxic cells group.

**Table 5:** The relative content of ROS in CNE-1 cells ($\overset{-}{x}\pm s$) %.

Groups	Relative content of ROS
Control	54.69 ± 3.21
Hypoxia	30.19 ± 2.69
Emodin	69.54 ± 3.69
2 Gy	117.71 ± 5.47
Hypoxia + 2 Gy	85.34 ± 5.84
Hypoxia + emodin	60.23 ± 5.96
Emodin + 2 Gy	201.41 ± 6.78^*⋆*^
Emodin + hypoxia + 2 Gy	154.91 ± 6.25^*⋆*^

^⋆^ *P* < 0.05, compared to the hypoxia + 2 Gy group.

**Table 6:** Maximum tumor diameter doubling time and tumor growth delay associated with different treatments ($\overset{-}{x}\pm s$, *n* = 6).

Group	Maximum tumor diameter doubling time/day	Tumor growth delay/day	Enhancement factor/%
Control group	17.02 ± 1.12	—	
Solvent group	17.14 ± 0.63	0.12	
Low-dose emodin (4 mg/kg)	17.20 ± 0.84	0.20	
High-dose emodin (12 mg/kg)	17.40 ± 1.01	0.40	
radiotherapy group	21.30 ± 1.14∗	4.28	
Low-dose emodin + 2 Gy	24.10 ± 0.72∗∗	6.90	1.612
High-dose emodin + 2 Gy	25.17 ± 0.64∗∗	8.15	1.904

^*^ *F* = 4.318, *P* < 0.05, Radiotherapy group compared to control group, ∗∗*F* = 2.163, *P* < 0.05, combined group versus simple radiotherapy group.

## Abbreviations

NPC: Nasopharyngeal carcinoma
DMF: Dose modification factors
HIF: Hypoxia-inducible factor
ROS: Reactive oxygen species
DEF: Dose enhancement factor
ODD: Oxygen-dependent degradation domain
ORP: Oxidation-reduction potential
PM: *Polygonum multiflorum*
NBS: Newborn bovine serum
DMSO: Dimethyl sulfoxide
PI: Propidium iodide
EF: Enhancement factor
ANOVA: Analysis of variance
*C*_*t*_: Threshold cycle
DCFH-DA: 2′,7′-dichlorofluorescin diacetate
FITC: Fluorescein isothiocyanate.
